# Elucidating the Role of Ce in Enhancing Phosphorus Poisoning Resistance of Pd-SSZ-13 for Passive NO*_x_* Adsorber

**DOI:** 10.3390/molecules31213899

**Published:** 2026-10-29

**Authors:** Dongdong Chen, Feng Liang, Zhenzhen Jia, Bo Zhang, Shasha Liu, Chihpeng Lin, Huibin Liu, Chungfu Huang, Xiang Li

**Affiliations:** 1 School of Environmental and Chemical Engineering, Zhaoqing University, Zhaoqing 526061, Chinajiazhenzhen@zqu.edu.cn (Z.J.); david_zhang200309@163.com (B.Z.); liushasha@zqu.edu.cn (S.L.); huangchongfu@zqu.edu.cn (C.H.); lixiang@zqu.edu.cn (X.L.); 2 National Engineering Laboratory for VOCs Pollution Control Technology and Equipment, Guangdong Provincial Key Laboratory of Atmospheric Environment and Pollution Control, School of Environment and Energy, South China University of Technology, Guangzhou 510006, China; 202410190538@mail.scut.edu.cn

**Keywords:** passive NO*_x_* adsorber, Pd-SSZ-13, Ce modification, P tolerance

## Abstract

Passive NO*_x_* adsorber (PNA) based on palladium-loaded small-pore SSZ-13 zeolite (Pd-SSZ-13) is attractive for mitigating cold-start NO*_x_* emissions from lean-burn engines, but its practical deployment is hampered by susceptibility to chemical poisons—most notably phosphorus (P) poisoning present in vehicle exhaust. In this work, cerium (Ce) is introduced for the first time as a promoter to enhance the P-tolerance of Pd-SSZ-13. Activity measurements show that, after exposure to P-containing conditions, the Ce-doped catalyst adsorbs ~47.9 µmol/g of NO*_x_*, which is markedly higher than that of the unmodified counterpart (~39.9 µmol/g). Comprehensive characterization (in situ DRIFTS, in situ UV-Vis, XPS, HAADF-STEM, and H_2_-TPR) reveals that Ce incorporation stabilizes the framework aluminum associated with Brønsted acid sites and markedly suppresses the deleterious interaction between P and active Pd^2+^ species, thereby attenuating P-induced deactivation. These findings demonstrate that Ce not only improves resistance of Pd-SSZ-13 to P poisoning but also extends its robustness toward other common poisons (e.g., alkali metals, sulfur species). Consequently, Ce-modified Pd-SSZ-13 emerges as a promising strategy for durable PNA catalysts in real-world exhaust after-treatment systems.

## 1. Introduction

As a rapidly burgeoning research frontier in automotive exhaust aftertreatment, PNA systems exhibit exceptional efficacy in suppressing low-temperature NO*_x_* slip during the transient cold-start phase of internal combustion engines, a critical operating window spanning ~100–200 s post-ignition [1,2]. To date, PNA formulation research has converged on small-pore CHA-type zeolite-supported Pd catalysts (e.g., Pd-SSZ-13), driven by two key merits of the CHA framework: first, its inherent hydrothermal stability and intrinsic resistance to chemical poisoning; second, its optimal NO*_x_* release performance at temperatures exceeding 300 °C [3,4,5]. Analogous to the majority of automotive aftertreatment catalysts, PNA are highly susceptible to chemical poisoning, a key failure mode that drastically curtails catalytic service lifetime [6,7]. Among exhaust-borne contaminants, phosphorus species derived from zinc dialkyldithiophosphate (ZDDP) additives in volatile engine lubricants and alkyl polyphosphoric acids in fuel are among the most ubiquitous catalytic poisons, capable of inducing irreversible deactivation of PNA systems [3,8,9,10,11]. Using Pd-SSZ-13 as a representative model system, phosphorus-induced deactivation can be attributed to two dominant mechanisms [10,11]: First, P species corrode framework Al sites in the CHA zeolite, triggering catastrophic framework collapse and subsequent pore blockage. Second, P species can also coordinate with isolated active Pd sites (i.e., Pd^2+^ and [Pd(OH)]^+^), driving the migration of these active sites from their native ion-exchange positions to the zeolite external surface, where they sinter and aggregate into bulk PdO_x_ phases, culminating in total loss of catalytic functionality.

Compared to conventional remediation strategies for zeolitic catalyst poisoning—such as high-temperature thermal decomposition and strong acid washing—chemical modification via rare-earth element (e.g., Ce) incorporation stands out as a far more promising approach, distinguished by its significantly reduced implementation cost and streamlined operational workflow [12,13,14,15]. For instance, Guo et al. [12] leveraged a suite of ex situ/in situ characterizations coupled with density functional theory (DFT) calculations to demonstrate that P species exhibit preferential binding affinity for Ce species, thereby conferring protection to active Fe sites, Brønsted acid sites, and framework Al in Fe-Beta zeolite. In a parallel study, Chen et al. [13] elucidated the mechanistic basis for the enhanced phosphorus resistance of Ce-modified Cu-SSZ-13 zeolite catalysts, identifying two distinct anti-poisoning pathways: On the one hand, Ce incorporation into and occupancy of 8-membered ring (8-MR) sites in the CHA framework drives the selective localization of active Cu^2+^ species at 6-membered ring (6-MR) positions, generating isolated Cu^2+^ sites with markedly improved phosphorus tolerance. On the other hand, Ce sites function as dedicated phosphorus capture receptors that preferentially adsorb phosphorus-based poisons, forming a protective shield around active Cu^2+^ sites to prevent deactivation and preserve catalytic activity. In related work, Xiao et al. [15] confirmed that Ce acts as a sacrificial site that preferentially binds P to form thermodynamically stable CePO_4_ phases, effectively shielding SCR-active Cu^2+^ centers and preserving the redox activity of copper sites in Cu-LTA zeolite. Notably, despite these demonstrated successes of Ce modification in anti-phosphorus poisoning for Fe- and Cu-based zeolite catalysts, no studies to date have explored the application of Ce doping to enhance the phosphorus tolerance of active Pd species in zeolitic PNA catalysts (e.g., Pd-SSZ-13). Given the proven anti-poisoning efficacy of Ce in analogous catalytic systems, Ce-mediated modification of Pd-SSZ-13 presents a highly promising strategy to suppress P-induced deactivation and extend the service lifetime of PNA catalysts.

In the present study, pristine Pd-SSZ-13 and its Ce-modified analogue (denoted as Ce-Pd-SSZ-13) were successfully synthesized. To simulate the phosphorus-induced deactivation process, the as-synthesized catalysts were subjected to phosphorus poisoning treatment via incipient wetness impregnation with di-Ammonium hydrogen phosphate ((NH_4_)_2_HPO_4_) serving as the phosphorus precursor. A comprehensive suite of complementary ex situ and in situ characterization techniques was employed to uncover the underlying anti-poisoning mechanisms, revealing that Ce incorporation via a simplified wet impregnation strategy can simultaneously reinforce the framework Al sites and strengthen the metal-support interactions between cationic Pd species and the CHA zeolitic framework. Consequently, surface-anchored Ce species form a robust protective shell on the external surface of the zeolite, which effectively anchors the CHA framework and stabilizes the active Pd^2+^ sites upon phosphorus exposure. This study establishes a fundamental theoretical framework for the rational design of phosphorus-tolerant PNA materials, providing a viable pathway for their practical deployment in automotive exhaust aftertreatment systems during the critical engine cold-start phase.

## 2. Results and Discussions

### 2.1. NO_x_ Adsorption/Desorption Performance over Pd-SSZ-13 Samples

**Figure 1:**
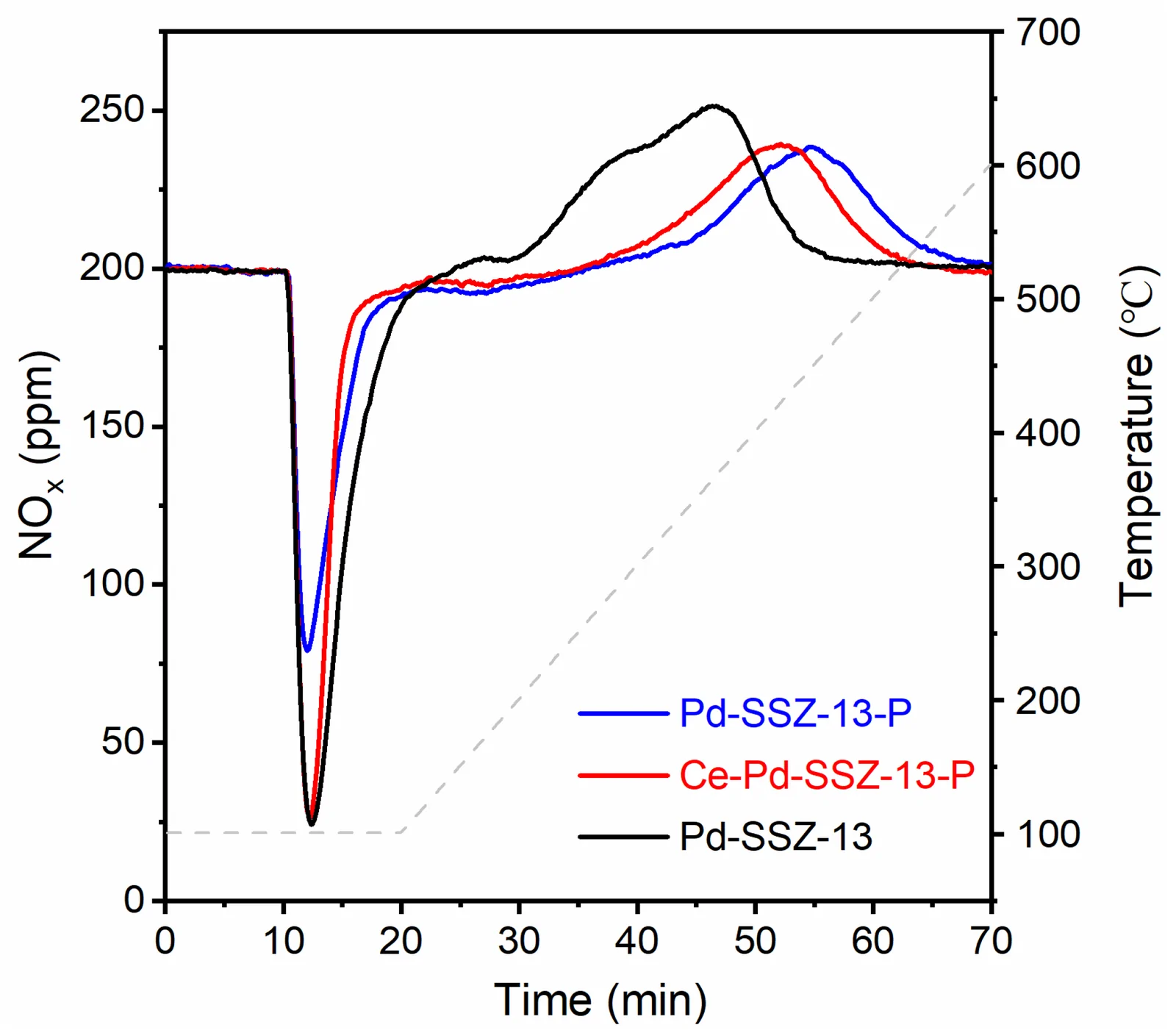
PNA performances of fresh Pd-SSZ-13, P-poisoned Pd-SSZ-13-P and Ce-Pd-SSZ-13-P catalysts. All samples were pre-treated at 500 °C before testing. Feed composition: 200 ppm NO*_x_*, 200 ppm CO, 3 vol.% H_2_O, 10 vol.% O_2_ in N_2_, with a total flow rate of 200 mL·min^−1^. Note: the dotted line in Figure 1 represents the real-time temperature during the adsorption and desorption process.

To elucidate the impact of Ce incorporation on the anti-poisoning performance of Pd-SSZ-13 when deployed as PNA catalyst, a systematic comparative evaluation of the NO*_x_* adsorption–desorption behaviors was conducted between unmodified and Ce-modified Pd-SSZ-13 samples. Given that water vapor and CO are ubiquitous constituents in diesel exhaust that profoundly modulate the NO*_x_* adsorption–desorption dynamics of PNA systems [1,16], 3 vol.% H_2_O and 200 ppm CO were spiked into the feed gas during performance testing to faithfully replicate authentic on-road operating conditions, thereby enhancing the ecological validity of the experimental design. With a fixed NO*_x_* inlet concentration of 200 ppm, the negative concentration deviations below 200 ppm observed during the initial 10–20 min of time-on-stream correspond to net NO*_x_* uptake by the catalyst, representing the NO*_x_* storage capacity of the sample during this period; conversely, the positive deviations exceeding 200 ppm detected after 20 min stem from the thermal desorption of previously stored NO*_x_* species. As depicted in Figure 1, the NO*_x_* storage capacity of the fresh Pd-SSZ-13 sample plummeted from 73.1 µmol/g to 39.9 µmol/g after exposure to phosphorus poisoning (denoted as Pd-SSZ-13-P), whereas the Ce-modified P-poisoned sample (Ce-Pd-SSZ-13-P) exhibited a markedly restored NO*_x_* storage capacity of 47.9 µmol/g. Consistent with prior literature reporting that the NO*_x_* storage performance of PNA under realistic operating conditions is intimately correlated with the population of redox-active Pd^2+^ sites hosted in the SSZ-13 zeolite framework [2,8,17], the present adsorption–desorption profiles demonstrate that P poisoning severely suppresses NO*_x_* storage capacity, while the Ce modification effectively attenuates this inhibitory effect to a substantial extent. Collectively, the results derived from Figure 1 and Supplementary Figure S1 support the reasonable inference that Ce incorporation alleviates the deleterious impact of P poisoning on the redox-active Pd species localized at cation exchange sites within the zeolite. The molecular-scale mechanism underlying Ce-mediated mitigation of P poisoning will be dissected in detail in the following section.

**Figure 2:**
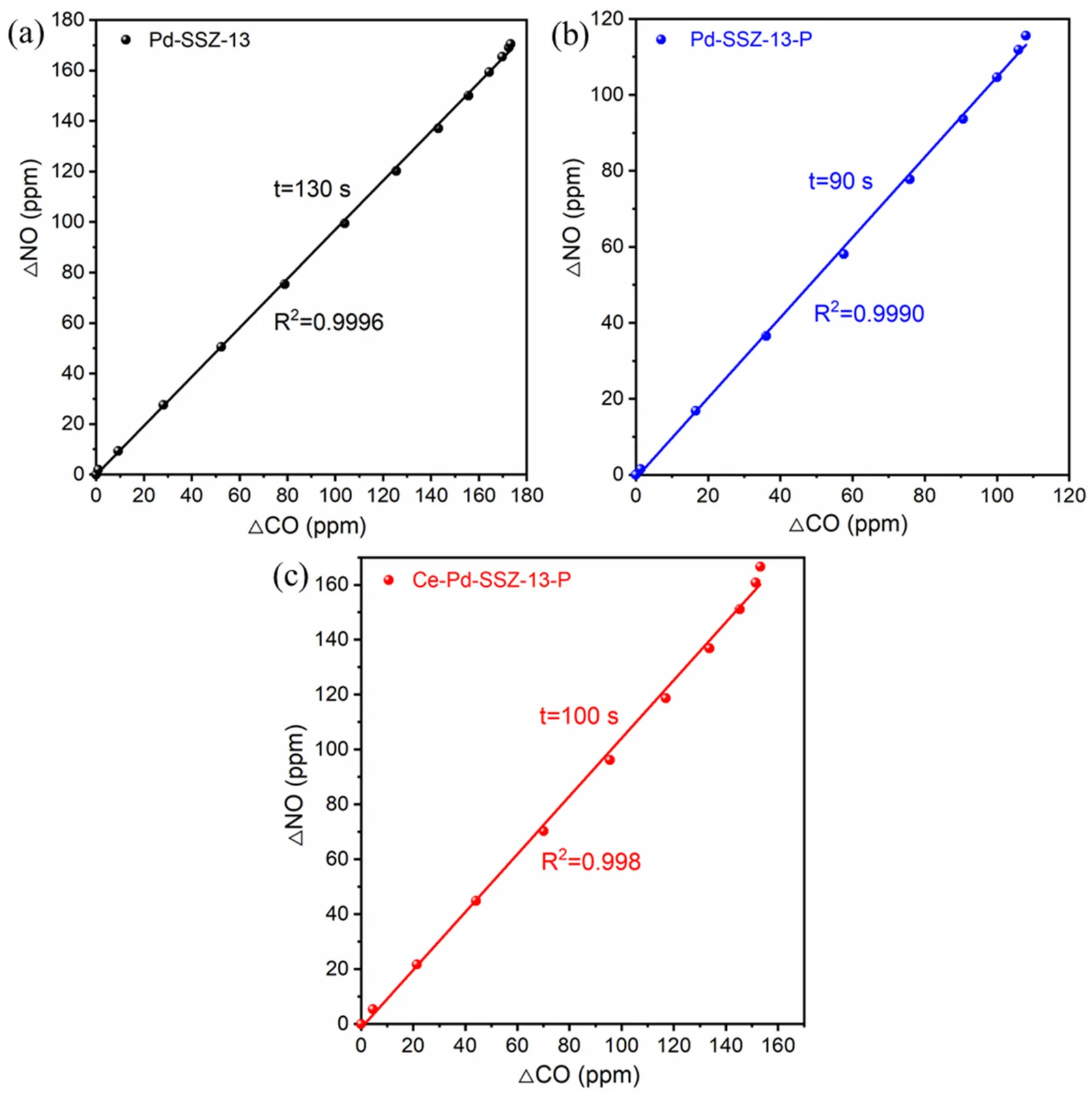
The correlation between the reduced concentrations of NO and CO (labeled as ΔNO and ΔCO) during the initial stage of NO*_x_* adsorption in PNA tests over Pd-SSZ-13 (**a**), Pd-SSZ-13-P (**b**) and Ce-Pd-SSZ-13-P (**c**). ΔNO (ΔCO) = *C*_0_ − *C_t_* (*C*_0_ and *C_t_* represent initial and real-time concentrations of NO or CO, respectively).

Prior literature has established that co-adsorption of NO and CO onto isolated Pd^2+^ sites under operando reaction conditions yields robust, stable carbonyl-nitrosyl adducts—specifically the catalytically relevant Pd^2+^(NO)(CO) species [2,18]. Building on this mechanistic foundation, our group’s earlier work has provided irrefutable evidence that CO and NO undergo stoichiometric 1:1 consumption during the early adsorption transient, a characteristic signature that definitively corroborates the formation of the catalytically active Pd^2+^(NO)(CO) intermediate [3,4,8]. To leverage this established framework, we conducted a semi-quantitative appraisal of the population of catalytically active ionic Pd^2+^ across the series of tested catalysts, derived from analysis of the temporal CO and NO consumption trajectories at the reaction onset; the corresponding trends are summarized in Figure 2. Figure 2 clearly reveals that substantially higher integrated CO and NO consumption is observed during PNA testing over Pd-SSZ-13, indicative of a markedly higher density of well-dispersed isolated Pd^2+^ sites in this parent material. Notably, P incorporation induces a pronounced depletion of accessible active Pd^2+^ sites, thereby suppressing the generation of the Pd^2+^(NO)(CO) intermediate. By contrast, Ce incorporation counteracts the deleterious site-blocking effect of P on Pd centers, restoring and even elevating the population of the Pd^2+^(NO)(CO) intermediate upon Ce modification, relative to the P-poisoned Pd-SSZ-13-P analogue. This mechanistic interpretation will be rigorously substantiated by a comprehensive battery of orthogonal characterization techniques in the ensuing sections of this work.

### 2.2. Characterizations of the Catalysts

Crystalline phase constitution, surface topographical features, and porous structural attributes of the as-synthesized zeolite specimens were comprehensively characterized via XRD, FE-SEM, and N_2_ physisorption analyses, with the corresponding results compiled in Figures S2–S4. As evidenced by the XRD patterns of all tested specimens (Figure S2), all samples exhibit the diffraction peaks characteristic of the CHA-type zeolite topology, verifying the crystallographic framework integrity of both Pd-SSZ-13. In comparison with the other two specimens, the Ce-Pd-SSZ-13-P sample shows a pronounced reduction in the intensities of the CHA-associated diffraction peaks, an observation primarily attributable to the deposition of dispersed CeO_x_ crystallites on the external surface of the zeolite support [18,19]. FE-SEM micrographs reveal that all as-prepared samples consist of well-defined cubic crystallites, and no perceptible modification in surface topography is observed either prior to or following P loading and Ce modification (Figure S3). N_2_ physisorption characterization results in Figure S4 further indicate that phosphorus poisoning triggers a marked decrease in the specific surface area of the Pd-SSZ-13 catalyst, which plummets from 527.16 to 454.24 m^2^/g. This pronounced structural degradation is predominantly attributable to two synergistic degradation mechanisms: (i) dealumination-induced collapse of the zeolite crystalline framework, arising from phosphorus species deposition within the micropores; and (ii) obstruction of microporous/mesoporous transport channels, caused by the thermodynamically driven agglomeration of encapsulated Pd active phases during the poisoning treatment. By contrast, the Ce-modified Pd-SSZ-13 catalyst retains a specific surface area of 508.10 m^2^/g even after undergoing the identical phosphorus poisoning treatment, which unequivocally validates that Ce modification can effectively mitigate the detrimental effects of phosphorus on both the zeolite support and atomically dispersed Pd species, thereby substantially attenuating overall catalyst deactivation.

**Figure 3:**
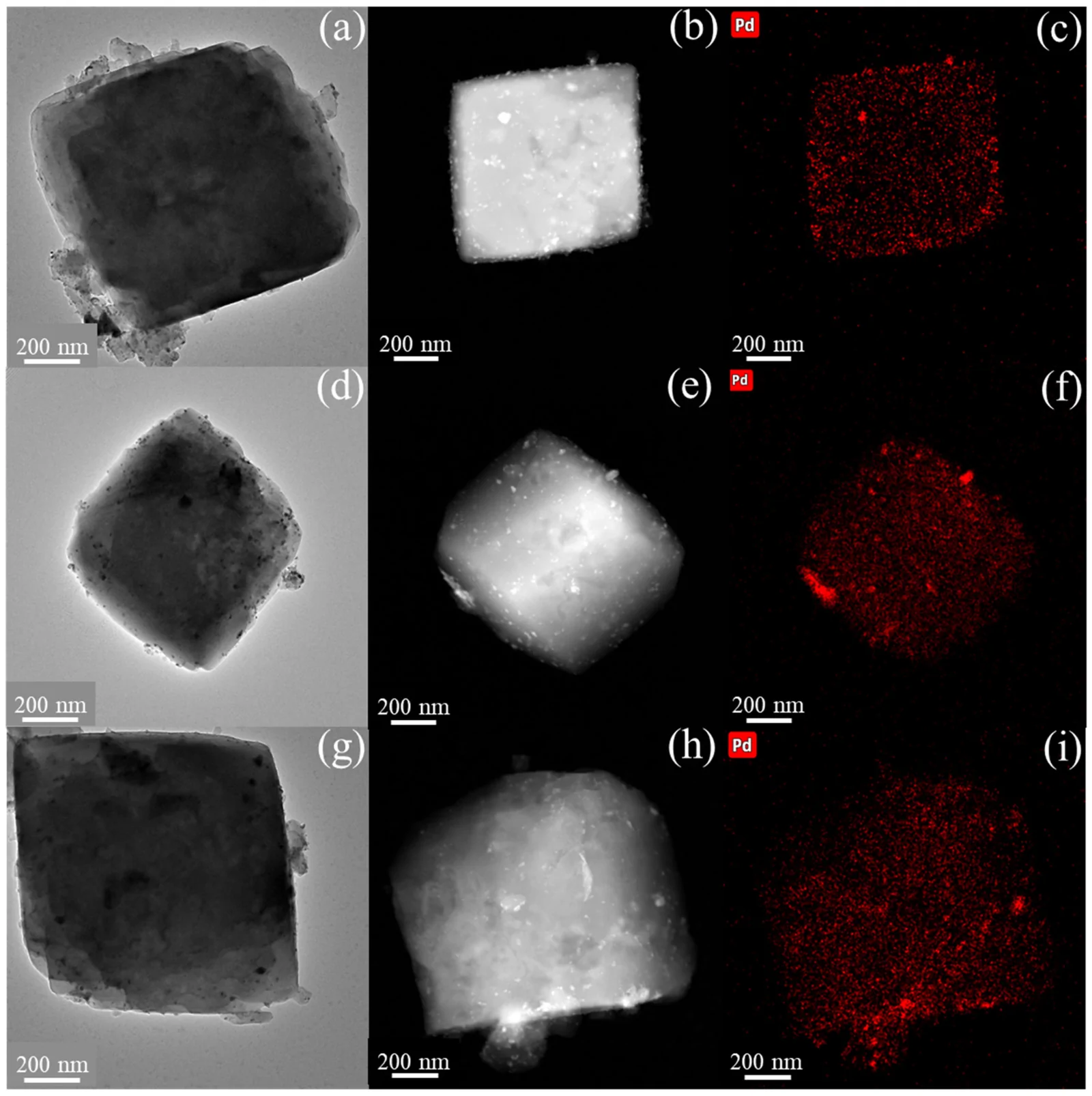
TEM (**a**,**d**,**g**), HAADF-STEM images (**b**,**e**,**h**), and (**c**,**f**,**i**) EDX mapping of Pd for the fresh Pd-SSZ-13 (panels (**a**–**c**)), P-poisoned Pd-SSZ-13 (panels (**d**–**f**)) and Ce- Pd-SSZ-13 samples (panels (**g**–**i**)).

STEM was harnessed to resolve the catalyst morphology at nanoscale precision. Representative low-magnification HAADF-STEM images, accompanied by quantitative EDS elemental maps, are displayed in Figure 3 and Figures S5–S7. In the pristine Pd-SSZ-13 sample (Figure 3a,b), virtually no discernible large particles are observed, indicating that the Pd species are homogeneously dispersed throughout the CHA framework. Upon exposure to phosphorus, however, pronounced Pd agglomeration emerges on the external zeolite surface, unequivocally indicating that P promotes the migration of cationic Pd^2+^ sites from the framework to the exterior, thereby driving the nucleation of bulk-like PdO_x_ aggregates (Figure 3d,e). For the Ce-modified analogue (P-poisoned Ce-Pd-SSZ-13; Figure 3g,h), the density of Pd nanoparticles on the zeolite exterior is markedly diminished, suggesting that Ce incorporation suppresses the conversion of isolated Pd^2+^ into PdO_x_ aggregate entities. Complementary EDS mapping (Figure 3c,f,i and Figures S5–S7) confirms uniform Si and Al distribution across all as-synthesized materials, whereas Pd exhibits conspicuous enrichment on the zeolite surface. Within the Pd-SSZ-13 system, Pd is well dispersed, with virtually no detectable aggregated regions, indicating that Pd predominantly exists in the ionic state (Figure 3c). Notably, while both Pd-SSZ-13 and Ce-Pd-SSZ-13 display Pd-rich domains after P treatment (Figure 3f,i), the extent of surface Pd aggregation is substantially attenuated in the Ce-containing sample, concomitant with a more uniform Pd distribution within the CHA lattice. This redistribution of Pd is further validated by CO pulse chemisorption. As illustrated in Figure S8, the P-laden Pd-SSZ-13 exhibits a reduced Pd dispersion of 15.9% relative to its unpoisoned counterpart (52.6%). Incorporation of Ce ameliorates the deleterious effect of P poisoning, restoring Pd dispersion to 24.3%. Collectively, these observations substantiate that Ce plays a pivotal role in mitigating P-induced Pd migration and agglomeration, thereby fostering a more homogeneous Pd^2+^ population confined within the zeolite matrix.

**Figure 4:**
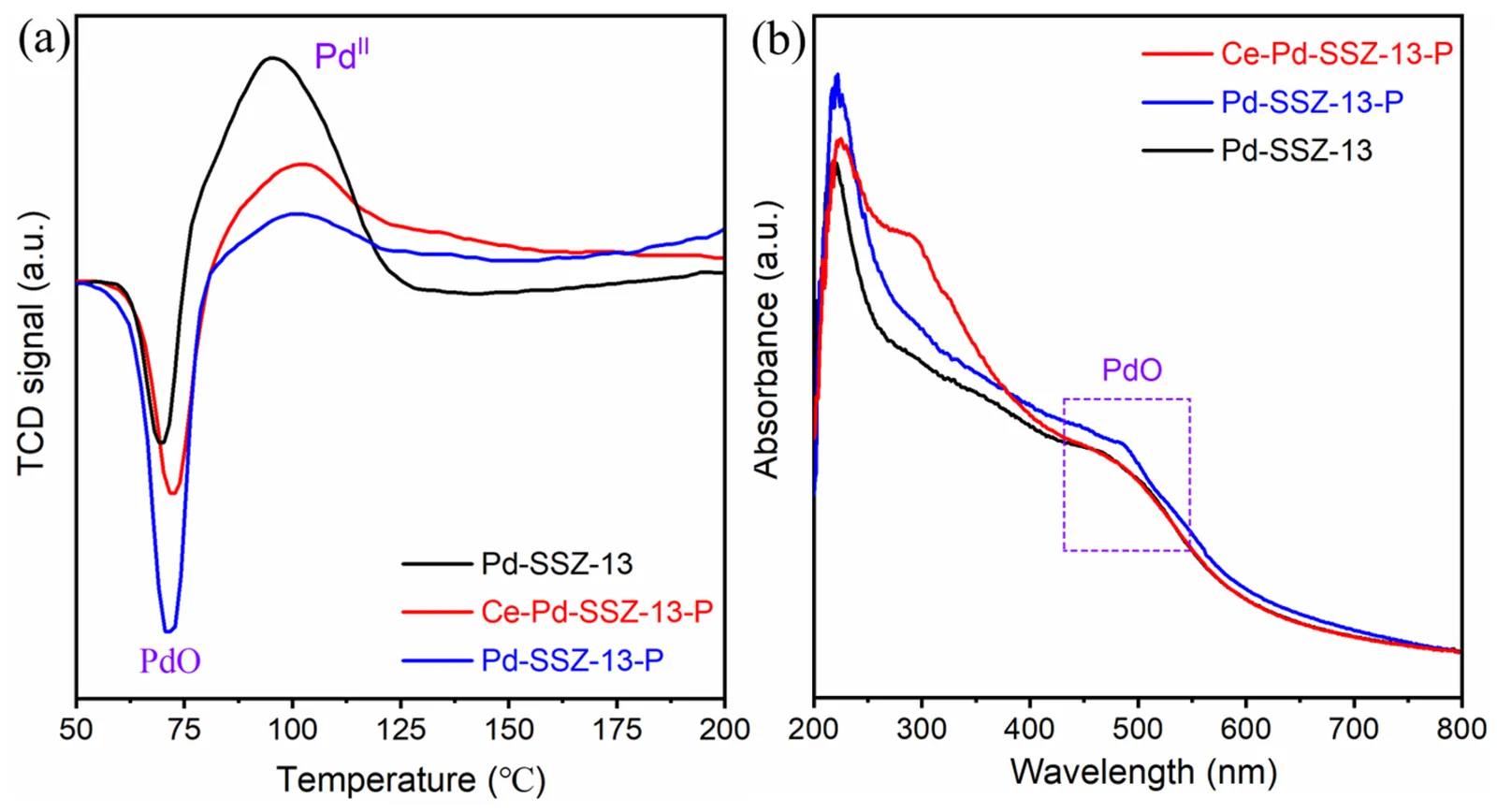
H_2_-TPR curves (**a**) and in situ UV-Vis spectra (**b**) of Pd-SSZ-13, Pd-SSZ-13-P and Ce-Pd-SSZ-13-P catalysts.

It is well-documented in the literature that PdO_x_ species and cationic Pd^2+^ exhibit distinct H_2_ consumption signatures across disparate temperature regimes, which underpins the widespread application of H_2_-TPR profiling for quantifying Pd^2+^ dispersion and tracking morphological evolution of PdO_x_ particulates in zeolite-supported catalytic systems [20,21,22]. As delineated in Figure 4a, the H_2_-TPR profile of the pristine Pd-SSZ-13 catalyst displays a pronounced, well-defined positive H_2_ consumption resonance spanning 90–110 °C; this signal is unambiguously assigned to the reductive conversion of isolated active Pd^2+^ moieties, providing definitive corroboration of the high prevalence of cationic Pd species in the fresh catalyst [3,22,23]. Notably, this characteristic peak undergoes a drastic attenuation in intensity following phosphorus loading in the P-poisoned Pd-SSZ-13-P sample, directly evidencing the severe depletion of active Pd^2+^ species induced by P exposure. Conversely, upon Ce incorporation, the intensity of the aforementioned Pd^2+^-derived peak is partially restored to an intermediate level between the pristine and P-poisoned samples, which directly corroborates that Ce can substantially suppress the phosphorus-induced deactivation of active cationic Pd^2+^ species, though complete recovery is not achieved. Concomitantly, the negative H_2_ signal (i.e., H_2_ release peak) observed at ~70 °C is assigned to the decomposition of palladium hydride (PdH_x_) phases, which are generated via a two-step process: first, the reductive conversion of bulk PdO_x_ to metallic Pd^0^ under H_2_ atmosphere, followed by the spontaneous hydrogenation of Pd^0^ to form PdH_x_ in the presence of excess H_2_ [8,22,23]. As can be discerned from the profile, the intensity of the Pd^2+^-derived reduction peak for the Ce-Pd-SSZ-13-P falls intermediate between those of the unpoisoned pristine Pd-SSZ-13 and the P-poisoned Pd-SSZ-13-P catalysts, which unambiguously confirms that Ce incorporation substantially suppresses the deleterious conversion of the active Pd^2+^ species into bulk PdO_x_ moieties triggered by phosphorus exposure. In situ UV-Vis spectroscopy (Figure 4b) was further used to verify the nature of species changes observed via H_2_-TPR, providing structural evidence for the aforementioned behavioral differences at the coordination level. It can be observed that fresh Pd-SSZ-13 catalyst displays a strong absorption band at ~230 nm assigned to the ligand-to-metal charge transfer (LMCT) between ionic Pd^2+^ and zeolite framework oxygen, directly proving Pd exists as highly dispersed isolated Pd^2+^ in the fresh sample [9]. By contrast, for the phosphorus-poisoned Pd-SSZ-13 sample, besides the peak at ~230 nm, a pronounced shoulder peak is observed at ~480 nm; this peak is assigned to the characteristic feature of aggregated PdO_x_, indicating that phosphorus deposition indeed induces the transformation of isolated Pd^2+^ into larger-sized PdO_x_ aggregates [9,21,24,25]. In contrast to the phosphorus-poisoned sample, following Ce modification, the 480 nm absorption band assigned to aggregated PdO_x_ disappears entirely, and a new broad absorption band emerges at ~300 nm, which is attributed to the LMCT between Ce^3+^ and the zeolite framework oxygen [13,15,19,26,27]. This indicates that Ce introduction effectively suppresses phosphorus-induced agglomeration of isolated Pd^2+^, preserves the majority of highly dispersed isolated Pd^2+^ active sites, and is consistent with the TPR observations of recovered low-temperature Pd^2+^ reduction peak intensity and attenuated PdH_x_ release signal.

**Figure 5:**
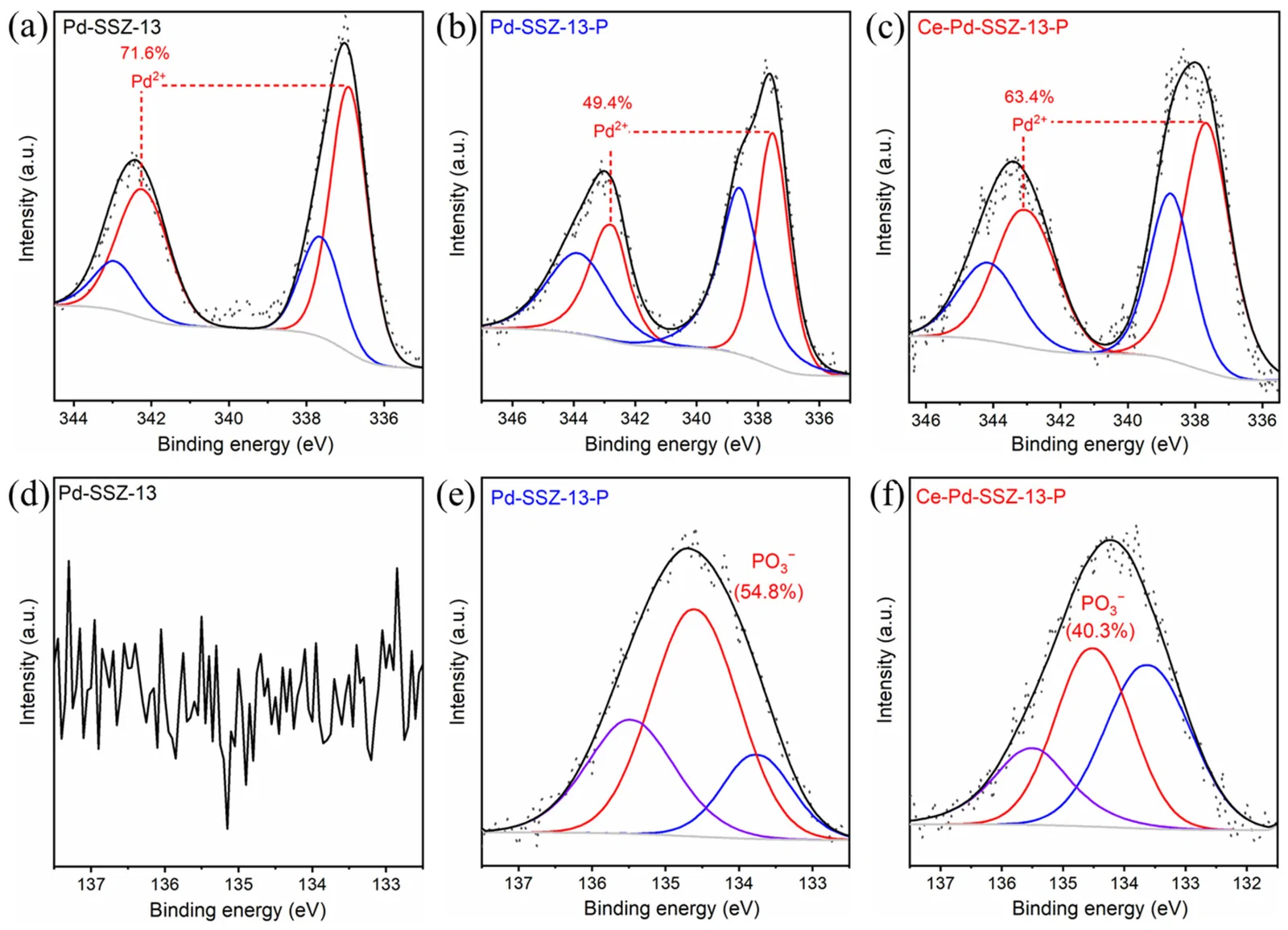
High-resolution XP Pd 3d and P 2p spectra of the Pd-SSZ-13 before (**a**,**d**) and after (**b**,**e**) P poisoning, and P-poisoned Ce-Pd-SSZ-13 catalyst (**c**,**f**). Note: the red and blue peaks in (**a**–**c**) are characteristic of Pd^4+^ and Pd^2+^, respectively; The blue, red, and violet peaks in (**e**,**f**) are characteristic of PO_4_^3−^, PO_3_^−^, and P_2_O_5_, respectively.

To systematically deconvolute the surface chemical speciation and valence state evolution of Pd active sites in as-synthesized Pd-SSZ-13 catalysts upon phosphorus poisoning and Ce modification, a comparative X-ray photoelectron spectroscopy (XPS) analysis was conducted, with the full set of deconvoluted spectra compiled in Figure 5a–f. The Pd 3d_5/2_ core-level spectra of Pd-SSZ-13 samples resolved two distinct, symmetric Gaussian-Lorentzian peaks centered at ~337.0 eV and ~337.8 eV, which were unambiguously assigned to Pd^2+^ and Pd^4+^ oxidation states, respectively [8,23]. Intriguingly, relative to Pd-SSZ-13-P, the binding energy of both the Pd 3d_5/2_ and Pd 3d_3/2_ core-level peaks exhibited a distinct positive shift. This spectral feature can be rigorously ascribed to the strong interfacial electronic interaction between PdO species and CeO_2_ domains anchored on the zeolitic support, leading to a pronounced depletion of electron density at Pd sites and consequently a more electron-deficient state of the Pd active centers. This observation further corroborates the strong synergistic effect between Pd and Ce components, aligning with the previously elucidated protective role of Ce against phosphorus-induced deactivation [28,29]. Quantitative comparative analysis of the Pd^2+^ peak areas between the pristine (non-poisoned) and phosphorus-poisoned Pd-SSZ-13 samples (Figure 5a,b) unveiled a pronounced 22.2% attenuation in the relative Pd^2+^ content in the poisoned sample, which dropped from 71.6% (fresh Pd-SSZ-13) to 49.4%. The concomitant increase in Pd^4+^ content, which can be rationalized as arising from PdO_2_ domains embedded in the bulk PdO_x_ phase dispersed on the zeolite external surface, further corroborates a higher overall loading of oxidized Pd species in the P-poisoned sample [8,9]. In stark contrast, Ce-Pd-SSZ-13-P sample exhibited a substantially augmented relative Pd^2+^ content of ~63.4%, which directly demonstrates that Ce exerts a pivotal protective effect against the phosphorus-induced conversion of atomically dispersed, catalytically active Pd^2+^ sites into inactive bulk PdO_x_ aggregates (Figure 5c). This conclusion is fully consistent with the complementary H_2_-TPR and in situ UV-Vis characterization results, which independently confirm the suppression of PdO_x_ aggregation upon Ce modification. Prior to phosphorus exposure, the pristine Pd-SSZ-13 catalyst exhibited no detectable P 2p signal in XPS spectrum, confirming the absence of phosphorus contamination before poisoning (Figure 5d). Subsequent deconvolution of the P 2p core-level spectra of the P-loaded samples (Figure 5e,f) identified three distinct phosphorus-containing species with binding energies (BEs) matching their standard assignments: phosphate (PO_4_^3−^) at 133.8 eV, metaphosphate (PO_3_^−^) at 134.6 eV, and phosphorus pentoxide (P_2_O_5_) at ~135.5 eV [8,30,31]. Notably, the intermediate BE peak corresponding to metaphosphate was identified as the dominant phosphorus species in both phosphorus-poisoned Pd-SSZ-13 samples, which is in excellent agreement with previously reported findings on phosphorus-poisoned automotive aftertreatment catalysts. Upon Ce modification, the relative proportion of metaphosphate decreased markedly from 54.8% to 40.3%, which can be ascribed to Ce’s steric and electronic blocking effect that impedes the nucleophilic attack of phosphorus-containing species on atomically dispersed [Pd(OH)]^+^ cationic sites, thereby suppressing the formation of condensed metaphosphate intermediates.

**Figure 6:**
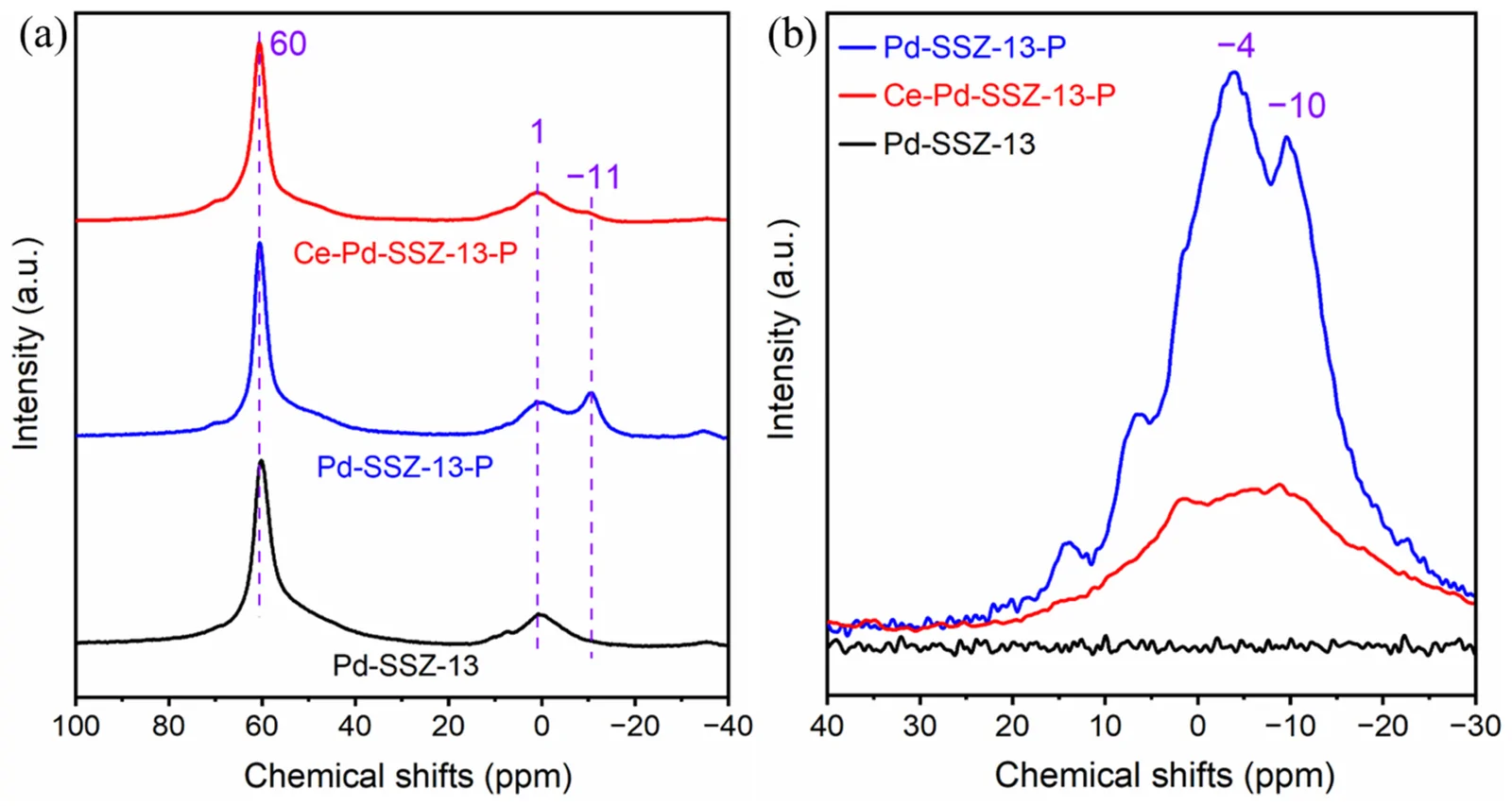
^27^Al (**a**) and ^31^P NMR (**b**) spectra of Pd-SSZ-13, Pd-SSZ-13-P and Ce-Pd-SSZ-13-P catalysts.

To elucidate subtle structural alterations in the zeolitic framework of Pd-SSZ-13 catalysts before and after P poisoning, solid-state ^27^Al and ^31^P MAS NMR spectroscopies were employed. As depicted in Figure 6a, the ^27^Al MAS NMR spectrum of the pristine Pd-SSZ-13 catalyst exhibits two well-resolved chemical shift resonances at 60 and 1 ppm, which are unambiguously assigned to tetrahedrally coordinated framework aluminum (AlO_4_) and octahedrally coordinated aluminum (AlO_6_), respectively [11,32]. Upon phosphorus poisoning, in addition to the aforementioned characteristic resonances at ~60 and 1 ppm, a prominent new peak emerges at −11 ppm. This spectral feature is congruent with the findings previously reported by Ilmasani et al. [11] and Wang et al. [22], and is predominantly ascribed to the formation of octahedrally coordinated aluminum nuclei within aluminum phosphate phases (i.e., amorphous Al-O-P species) generated via the reaction of phosphorus with framework aluminum [28]. Following Ce modification, the −11 ppm resonance is nearly completely extinguished, demonstrating that Ce incorporation efficiently impedes the poisoning of aluminum sites by phosphorus species, thereby conferring robust protection to framework aluminum against phosphorus-induced degradation [33]. The ^31^P MAS NMR spectra of the catalysts were shown in Figure 6b. For the pristine Pd-SSZ-13 catalyst prior to phosphorus poisoning, no discernible ^31^P resonance is detected, whereas distinct, well-defined peaks are observed for the two phosphorus-poisoned samples. The resonance at −4 ppm is assigned to low-condensation, short-chain polyphosphate species, which exist as weakly bound anionic phosphate clusters confined within the zeolite pore network [24,34]. In contrast, the resonance at −10 ppm is unambiguously attributed to P-O-Pd covalent linkages [8,22], providing direct evidence for robust coordinative interactions between phosphorus moieties and catalytically active Pd sites, which stem from the agglomeration of interfacial Pd-P species. Consistent with this spectral assignment, two well-resolved, distinct characteristic resonances at −4 and −10 ppm are clearly discernible in the ^31^P MAS NMR spectrum of the phosphorus-poisoned Pd-SSZ-13-P catalyst. In stark contrast, only a single broadened, symmetric resonance centered at −4 ppm is detected for the phosphorus-poisoned Ce-Pd-SSZ-13 analogue, with the −10 ppm signal being completely absent. This marked spectral discrepancy directly corroborates that Ce incorporation effectively attenuates the interaction between P and Pd, thereby suppressing the deleterious poisoning effect of phosphorus on catalytically active Pd sites, and ultimately alleviating catalyst deactivation triggered by phosphorus poisoning. Collectively, these solid-state NMR results provide robust evidence that Ce modification effectively suppresses the interactions between phosphorus species and both active Pd species and framework Al sites in the Pd-SSZ-13 catalyst.

**Figure 7:**
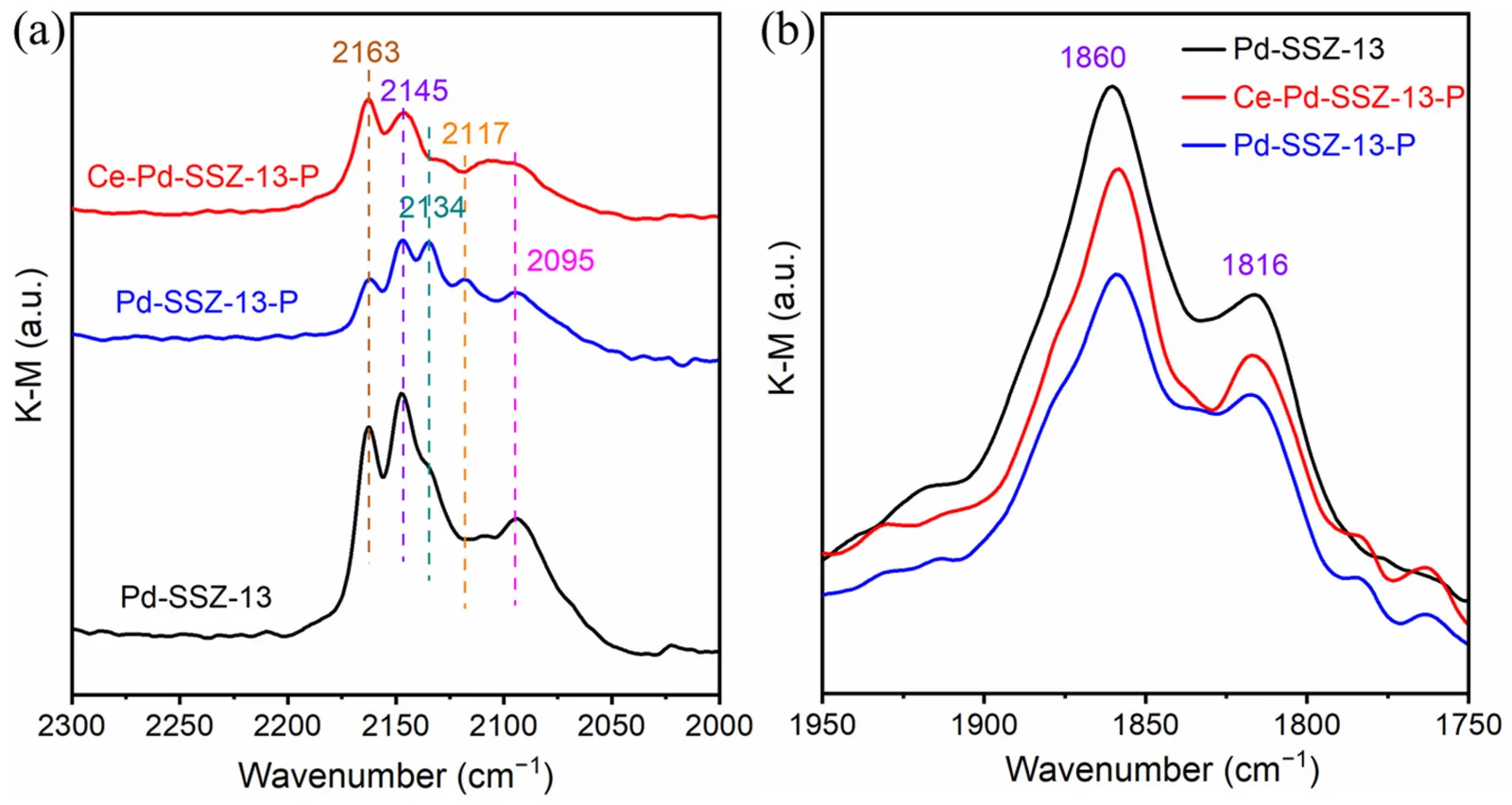
CO-DRIFTS (**a**) and NO-DRIFTS (**b**) profiles of Pd-SSZ-13, Pd-SSZ-13-P and Ce-Pd-SSZ-13-P catalysts.

An in situ DRIFTS study was conducted to further obtain the distribution of Pd species in non-poisoned and poisoned catalysts. Figure 7a shows the DRIFTS in the range of 1750–1950 cm^−1^ of the as-prepared three samples using NO as a probe molecule at 100 °C. An intense band at 1860 cm^−1^ and a feeble shoulder at 1816 cm^−1^ were observed, and they can be attributed to nitrosyl complexes adhering to two types of cationic Pd sites, i.e., Pd^2+^ in 6MR and [Pd(OH)]^+^ in 8MR, respectively. It is clear that the band intensities at 1860 and 1816 cm^−1^ significantly diminished after P poisoning. However, Ce modification considerably alleviated this deactivation effect. NO DRIFTS analysis reveals that Ce enhances the interaction between Pd and the zeolite framework, thereby reinforcing the stability of the active Pd species. In addition, Pd in zeolites (e.g., Pd-SSZ-13) can transition between highly dispersed and aggregated states depending on the reaction conditions, and this shift can be distinguished through in situ DRIFTS using CO as a molecular probe. Figure 7b shows the DRIFTS in the range of 2000–2300 cm^−1^ using CO as a probe molecule at 20 °C. Multiple characteristic bands appeared after exposure to CO; the bands at 2163 and 2148 cm^−1^ are attributed to Pd^2+^(CO)_2_ and [Pd(OH)]^+^(CO), respectively, corresponding to CO adsorbed on Pd^2+^ sites within 6MR and [Pd(OH)]^+^ within 8MR [35,36]. The peaks at 2134 and 2117 cm^−1^ corresponding to two mono-carbonyl species on Pd^+^, while the peak at 2095 cm^−1^ is attributed to CO adsorption on [Pd(OH)]^+^ [20,22,24,37]. It can be observed that for the Pd-SSZ-13 catalyst without P poisoning, the peaks at 2163 and 2145 cm^−1^ are its dominant characteristic peaks, indicating that Pd species in this system exist predominantly as isolated Pd^2+^ and [Pd(OH)]^+^. Following P poisoning of the Pd-SSZ-13 catalyst, the characteristic peaks associated with framework-confined Pd active sites undergo a marked attenuation in intensity. Complementarily, in congruence with the in situ spectroscopic observations reported by Wang et al. [22], we observed that P poisoning induces an antagonistic shift in CO adsorption profiles: the bands at ~2163 and 2145 cm^−1^ assigned to linearly adsorbed CO on isolated redox-active Pd^2+^ cations exhibit pronounced attenuation, whereas the bands at ~2134 and 2117 cm^−1^ corresponding to CO adsorbates on labile Pd^+^ moieties stabilized at low-coordination Pd-O_x_ ensembles show significant augmentation. This spectral variation unambiguously confirms that phosphorus exposure triggers spatially confined migration and concomitant structural restructuring of the active cationic Pd^2+^ species, which collectively underlies the observed phosphorus-induced catalytic deactivation. In contrast, the Ce-modified Pd-SSZ-13 sample exhibits stronger Pd-related characteristic peak intensities after exposure to P poisoning, especially for the peak at 2163 cm^−1^, indicating that a larger amount of active Pd^2+^ species is retained.

**Figure 8:**
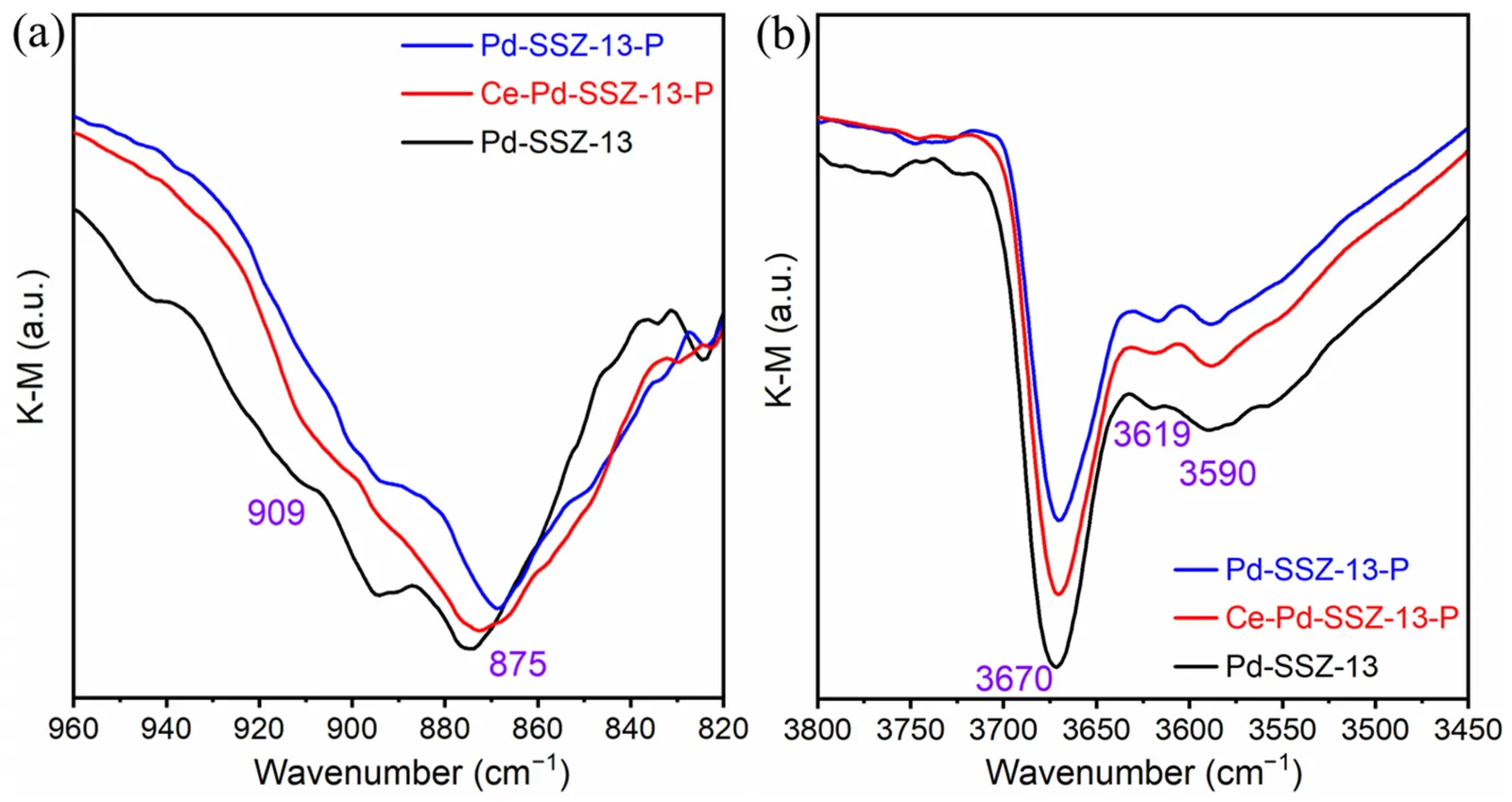
NH_3_-DRIFTS profiles of Pd-SSZ-13, Pd-SSZ-13-P and Ce-Pd-SSZ-13-P catalysts: (**a**) T–O–T regions; (**b**) OH vibrational region.

In situ NH_3_-DRIFTS serves as an indispensable characterization modality for elucidating the interaction between metal cations (e.g., Pd and Cu) and the zeolite framework [8,38,39,40]. A wealth of prior studies have corroborated that the incorporation of ion-exchanged metal cations perturbs the periodic T-O-T vibrational modes of the zeolite lattice; this perturbation can, however, be partially offset by the preferential adsorption of probe NH_3_ molecules onto coordinatively unsaturated active metal sites [3,8,39,40,41]. When NH_3_ adsorbs onto Pd^2+^ sites, the coordination interaction between Pd^2+^ and the zeolite framework is attenuated, generating two distinct negative absorption features in the 1000–850 cm^−1^ spectral region of the differential spectra [3,8,40,41]. These negative bands originate from the disruption of framework vibrational modes induced by two distinct Pd^2+^-derived species (i.e., Pd^2+^ and [Pd(OH)]^+^) anchored to the framework [41]. As illustrated in the in situ NH_3_-DRIFTS spectra in Figure 8a, two pronounced negative bands centered at ~875 and 909 cm^−1^ are detected for fresh Pd-SSZ-13 prior to phosphorus poisoning, which are respectively assigned to isolated monomeric Pd^2+^ and [Pd(OH)]^+^ species residing within the CHA-type zeolite cages [41]. After phosphorus poisoning, the characteristic [Pd(OH)]^+^ band at ~909 cm^−1^ is almost completely suppressed, while the intensity of the Pd^2+^-associated band at ~875 cm^−1^ also exhibits marked attenuation. In contrast, for the Ce-Pd-SSZ-13-P catalyst, a distinct band at ~908 cm^−1^ remains clearly observable, and the extent of intensity reduction for the ~872 cm^−1^ band is significantly smaller than that of the unmodified Pd-SSZ-13-P counterpart. This trend aligns with the well-acknowledged fact that [Pd(OH)]^+^ species confined in the 8MR pore windows of CHA zeolites are far more susceptible to phosphorus poisoning than Pd^2+^ species trapped in the 6MR; additionally, [Pd(OH)]^+^ is more prone to agglomerate into bulk PdO_x_ phases under hydrothermal reaction conditions [8,10,11]. Furthermore, Figure 8b displays the OH vibrational region of the three samples. The two bands at 3590 and 3620 cm^−1^, which are attributed to Brønsted acidic hydroxyl groups (i.e., Si–O(H)–Al) that are protonated by adsorbed NH_3_ [38,39,42], leading to their apparent consumption in the differential spectra. The band at 3670 cm^−1^ is assigned to the framework Al–OH stretching vibration [10,35]. Notably, the integrated intensity of Al–OH and the Brønsted acid site-associated bands decreases markedly after phosphorus poisoning, indicating severe framework dealumination; this detrimental structural degradation is, however, substantially suppressed upon Ce modification of the zeolite. Collectively, the complementary in situ NH_3_-DRIFTS results in Figure 8 demonstrate that the Ce-modified Pd-SSZ-13 catalyst exhibits vastly superior retention of both framework Brønsted acid sites and active Pd^2+^ species relative to the unmodified Pd-SSZ-13 analogue after phosphorus poisoning. These results indicate that Ce modification also exerts a dual protective function: on the one hand, it stabilizes framework Al atoms and inhibits the dealumination process, preserving the intrinsic acidity of the zeolite support; on the other hand, it strengthens the anchoring interaction between the zeolite framework and active Pd^2+^ sites, preventing the aggregation and leaching of active Pd species.

## 3. Experimental Section

### 3.1. Catalyst Synthesis

The as-received proton-form SSZ-13 zeolite (SiO_2_/Al_2_O_3_ molar ratio = 12) was sourced from Zhuoran Environmental Protection (Dalian) Co., Ltd. (Dalian, China). Palladium nitrate dihydrate (Pd(NO_3_)_2_·2H_2_O, Pd ≥ 39.0 wt.%), cerium nitrate hexahydrate (Ce(NO_3_)_3_·6H_2_O), ammonium sulfate ((NH_4_)_2_SO_4_) and (NH_4_)_2_HPO_4_ were procured from Shanghai Aladdin Biochemical Technology Co., Ltd. (Shanghai, China). All reagents were employed without further purification.

Initially, the SSZ-13 sample purchased was subjected to ion exchange in a 1.0 M solution of (NH_4_)_2_SO_4_ at room temperature for one hour according to the reported method [43]. Post ion exchange, the suspension was vacuum-filtered, the resulting solid was exhaustively rinsed with deionized water, and then dried at 200 °C to afford ammonium-form SSZ-13 (denoted as NH_4_^+^-SSZ-13). Next, the as-synthesized NH_4_^+^-SSZ-13 was impregnated with an aqueous solution of Pd(NO_3_)_2_·2H_2_O to achieve a Pd loading of 1 wt.%, followed by continuous stirring at 80 °C for 24 h. To maximize the population of ionic Pd species anchored on the zeolite support, the ion exchange step was repeated twice. After ion exchange treatment, the sample was recovered by filtration, rinsed repeatedly with deionized water, and subsequently calcined at 550 °C for 6 h under static air to yield the Pd-SSZ-13 catalyst. For the synthesis of Ce-Pd-SSZ-13, according to the work by Guo et al. [12], a moderate Ce content (mass fraction < 5 wt.%) can effectively enhance the phosphorus poisoning resistance of the catalyst, while excessive Ce loading (mass fraction > 5 wt.%) instead causes pore blockage in zeolite catalysts, leading to catalytic activity degradation. Therefore, a moderate Ce loading (i.e., 3 wt.%) was selected for the experimental design of this work. Specifically, 0.093 g of Ce(NO_3_)_3_·6H_2_O was dissolved in 1 mL of ultrapure water under constant stirring to prepare the Ce precursor solution (labeled as solution A). 1 g of Pd-SSZ-13 was then added to solution A, and the mixture was stirred manually for 20 min at ambient temperature. The resulting solid was then aged under dark conditions for 24 h, dried in an oven at 80 °C, ground thoroughly into fine powder, and finally calcined in a tubular furnace at 550 °C for 5 h to afford the target Ce-Pd-SSZ-13 catalyst.

In the literature, (NH_4_)_2_HPO_4_ is commonly used as the phosphorus precursor, with phosphorus loadings typically ranging from 0.2 to 0.8 mmol g^−1^ [8,10,11,30]. Specifically, aqueous (NH_4_)_2_HPO_4_ solution was added to a crucible containing 2 g of Ce-Pd-SSZ-13 or Pd-SSZ-13 powders to achieve a targeted phosphorus loading of 0.5 mmol/g in this work, and the mixture was stirred for 10 min. The crucible was then placed in an oven for 24 h at room temperature. Subsequently, the P-loaded samples were dried at 60 °C for 2 h, followed by drying at 110 °C for 15 h. Finally, the impregnated samples were calcined at 550 °C for 4 h in flowing air in a furnace. The resulting P-impregnated Ce-Pd-SSZ-13 and Pd-SSZ-13 samples were designated as Ce-Pd-SSZ-13-P and Pd-SSZ-13-P, respectively.

### 3.2. Catalyst Characterization

The physicochemical properties of the as-synthesized zeolite catalysts were systematically characterized via a suite of advanced analytical techniques, with specific experimental protocols detailed as follows: Powder X-ray diffraction (XRD) patterns of the zeolite specimens were acquired on a Rigaku Ultima IV advanced diffractometer (Rigaku, Tokyo, Japan) employing Cu Kα monochromatic radiation (operating parameters: tube voltage = 40 kV, tube current = 40 mA, wavelength λ = 1.5406 Å), with a continuous scanning rate of 10°·min^−1^ over the 2θ range of 5–50°. The textural properties, including specific surface area, pore volume and pore size distribution of the catalysts, were determined via nitrogen physisorption isotherms at 77 K on a Micromeritics ASAP 2020 analyzer (Norcross, GA, USA), with data processed according to the Brunauer–Emmett–Teller (BET) model and Barrett–Joyner–Halenda (BJH) algorithm. Hydrogen temperature-programmed reduction (H_2_-TPR) and CO pulse chemisorption experiments were performed on a BSD-Chem C200 automated chemisorption platform (BeiShiDe, Beijing, China). For each H_2_-TPR test, ~50 mg of calcined zeolite sample was loaded into a U-shaped quartz reactor, and the system temperature was ramped from ambient temperature to 850 °C at a heating rate of 10 °C·min^−1^ under a continuous flow of 10 vol.% H_2_/Ar (30 mL·min^−1^) as the reducing carrier. For CO pulse chemisorption assays, an equivalent sample mass of ~50 mg was utilized per run. Prior to probe molecule introduction, the sample underwent in situ reductive pretreatment at 200 °C for 2 h under 10 vol.% H_2_/Ar (30 mL·min^−1^), followed by cooling to the target adsorption temperature of 45 °C under the same gas flow. Subsequently, pulsed injections of 10 vol.% CO/He (30 mL·min^−1^) were administered to the pre-reduced Pd-SSZ-13 sample until adsorption saturation, with downstream CO concentration tracked in real time via a thermal conductivity detector (TCD) to quantify the amount of adsorbed CO. The morphological features of the zeolite samples were examined by ultra-high-resolution field-emission scanning electron microscopy (FE-SEM) on a Hitachi SU8600 instrument (Hitachi, Tokyo, Japan). High-angle annular dark-field scanning transmission electron microscopy (HAADF-STEM) images and corresponding energy-dispersive X-ray (EDX) elemental mappings were acquired on a FEI Talos F200X transmission electron microscope (Thermo Fisher Scientific, Waltham, MA, USA) at an accelerating voltage of 200 kV. The surface chemical composition and oxidation state of metal species on the zeolite samples were analyzed by X-ray photoelectron spectroscopy (XPS) on a Thermo Fisher Scientific ESCALAB 250 system (ESCALAB 250, Thermo Fisher Scientific, Waltham, MA, USA), employing a monochromatic Al Kα X-ray excitation source (photon energy hν = 1486.8 eV). Solid-state ^27^Al and ^31^P magic-angle spinning nuclear magnetic resonance (MAS NMR) spectra were recorded on a Bruker Avance III 600 MHz spectrometer (Bruker, Karlsruhe, Germany) equipped with a 4.0 mm cross-polarization (CP) MAS probe, with the MAS rotation frequency precisely calibrated to 12 kHz to eliminate line broadening induced by anisotropic dipolar interactions. For chemical shift calibration, 1 M Al(NO_3_)_3_ aqueous solution and (NH_4_)_2_HPO_4_ solid were used as external references for ^27^Al and ^31^P NMR, respectively. In situ diffuse reflectance ultraviolet–visible (UV-Vis) spectra were collected on a PerkinElmer Lambda 650 spectrometer (PerkinElmer, Waltham, MA, USA) integrated with a diffuse reflectance accessory consistent with the configuration employed for in situ diffuse reflectance infrared Fourier transform spectroscopy (DRIFTS) measurements. For in situ UV-Vis characterization, 50 mg of Pd-SSZ-13 zeolite sample was loaded into a Harrick high-temperature in situ reaction chamber coupled to a computerized gas dosing system. The sample was first activated at 500 °C for 1 h under a 21 vol.% O_2_ flow (50 mL·min^−1^) to remove surface adsorbates, then cooled to 30 °C under the same O_2_ flow. The DR UV-Vis spectra of the activated sample were then recorded in the wavelength range of 200–800 nm with a spectral interval of 2 nm. The background spectrum was collected over bare H-SSZ-13 support under 50 mL·min^−1^ N_2_ flow at 30 °C, and was subsequently subtracted from all Pd-SSZ-13 sample spectra to eliminate support contribution. In situ DRIFTS measurements were conducted on a Bruker INVENIO·R spectrometer (Bruker, Karlsruhe, Germany) coupled with a Harrick Praying Mantis diffuse reflection accessory and a high-temperature reaction chamber sealed with a ZnSe optical dome under controlled gas flow. All DRIFTS spectra were collected in the wavenumber range of 4000–600 min^−1^ with a spectral resolution of 2 min^−1^ and 64 cumulative scans to improve signal-to-noise ratio. To linearize the correlation between surface adsorbate coverage and band intensity, the raw interferogram signals were converted via the Kubelka–Munk (KM) function. Prior to each DRIFTS test, the sample was pretreated at 500 °C for 1 h under static air to remove residual moisture and surface contaminants.

### 3.3. Catalyst Evaluation

The NO adsorption–desorption behaviors of the as-synthesized catalysts were systematically characterized in a continuous-flow tubular quartz reactor. For each replicate experiment, 100 mg of catalyst (40–60 mesh) was loaded into the isothermal zone of the reactor to guarantee uniform gas flow and minimize axial dispersion. Prior to the NO test, the catalyst bed was activated by heating to 500 °C for 30 min under a gas stream containing 10 vol.% O_2_ balanced with N_2_. After activation, the temperature was lowered naturally to 100 °C while maintaining the same gas composition. Subsequently, the reaction feed consisting of 200 ppm NO*_x_*, 200 ppm CO, 3 vol.% H_2_O and 10 vol.% O_2_ (balanced with N_2_) was introduced at a total volumetric flow rate of 200 mL min^−1^. The system was held at 100 °C for approximately 10 min to allow NO uptake on the catalyst surface. Thereafter, the furnace temperature was ramped to 600 °C at a rate of 10 °C min^−1^ to probe the thermal desorption of adsorbed NO*_x_* species. Throughout the experiment, the effluent gas composition was monitored in real time using an online Fourier-transform infrared spectrometer (MKS MultiGas 2030, Andover, MA, USA) equipped with a heated gas cell maintained at 191 °C to prevent condensation of reactive intermediates. The acquired FTIR spectra were deconvoluted to quantify the concentrations of NO, NO_2_, CO and H_2_O during both adsorption and desorption steps.

## 4. Conclusions

The P poisoning resistance of pristine Pd-SSZ-13 and Ce-modified Pd-SSZ-13 was systematically investigated via simulated P poisoning protocols implemented through a controlled wet impregnation strategy, and the low-temperature NO*_x_* adsorption performance of the resultant samples was rigorously evaluated. Results demonstrate that Ce-modified Pd-SSZ-13 exhibits higher efficacy following P poisoning exposure. Given the limited number of samples included in the current design of experiments, the effects of Ce on storage capacity and poisoning resistance may not be fully separated and could be partially confounded. Although the comprehensive analysis of the samples provides valuable correlations, additional experiments involving a fully orthogonal design across all relevant variables would be beneficial for further clarifying the causal relationships. Nevertheless, in this work, we successfully employed a multimodal characterization toolkit comprising ex situ physicochemical analyses and state-of-the-art in situ spectroscopic techniques, including UV–Vis and DRIFTS. The results revealed that Ce incorporation not only stabilizes the zeolite framework by inhibiting the coordination of P species with framework Al sites, including Brønsted acid sites, but also mitigates the P-induced deactivation of Pd^2+^ active centers. These findings establish a robust rational design paradigm for engineering next-generation P-poisoning-resistant Pd/zeolite catalysts, marking a landmark advancement for the translational deployment of such PNA catalysts in low-temperature NO*_x_* abatement applications.

## Data Availability

The original contributions presented in this study are included in the article/Supplementary Materials. Further inquiries can be directed to the corresponding authors.

## Supplementary Materials

The following supporting information can be downloaded at https://www.mdpi.com/article/10.3390/molecules31213899/s1. Figure S1: Concentration profiles of CO during PNA tests over Pd-SSZ-13 catalysts; Figure S2: XRD patterns of P-poisoned Ce-Pd-SSZ-13, and Pd-SSZ-13 catalysts before and after P poisoning; Figure S3: Representative FE-SEM images of Pd-SSZ-13 (a), Pd-SSZ-13-P (b), and Ce-Pd-SSZ-13-P (c); Figure S4: N_2_ adsorption–desorption isotherms of Pd-SSZ-13, Pd-SSZ-13-P and Pd-SSZ-13-P catalysts; Figure S5: EDX mappings (Al, O, Si) of Pd-SSZ-13 sample; Figure S6: EDX mappings (Al, O, Si, P) of Pd-SSZ-13-P sample; Figure S7: EDX mappings (Al, O, Si, P, Ce) of Ce-Pd-SSZ-13-P sample; Figure S8: The profiles of CO pulse adsorption for the Pd-SSZ-13(a), P-poisoned Pd-SSZ-13-P (b) and Ce-Pd-SSZ-13-P (c) catalysts. Adsorbed CO amount and Pd dispersion for Pd-SSZ-13 samples as determined by pulse CO adsorption experiments. A CO/Pd stoichiometry of 1 was adopted to calculate the Pd dispersion. Reference [44] is cited in Supplementary Materials.
