# Supplementary material for: Elucidating the Role of Ce in Enhancing Phosphorus Poisoning Resistance of Pd-SSZ-13 for Passive NOx Adsorber
Source: Molecules. 2026 Oct 29;31(21):3899. doi: 10.3390/molecules31213899 (PMC13765496; doi:10.3390/molecules31213899)
Supplement: Supplementary file 1 [file molecules-31-03899-s001.zip › molecules-4595474-supplementary.pdf]

Supporting Information

Elucidating the Role of Ce in Enhancing Phosphorus Poisoning Resistance of Pd-SSZ-13 for Passive NO_x Adsorber

Dongdong Chen[1,*], Feng Liang[1], Zhenzhen Jia[1], Bo Zhang[1], Shasha Liu[1], Chihpeng Lin[1,*], Huibin Liu[2], Chungfu Huang[1] and Xiang Li[1]

1 School of Environmental and Chemical Engineering, Zhaoqing University, Zhaoqing 526061, China

2 National Engineering Laboratory for VOCs Pollution Control Technology and Equipment, Guangdong Provincial Key Laboratory of Atmospheric Environment and Pollution Control, School of Environment and Energy, South China University of Technology, 510006 Guangzhou, China

* Correspondence: chendongdong@zqu.edu.cn; ftirlin@foxmail.com

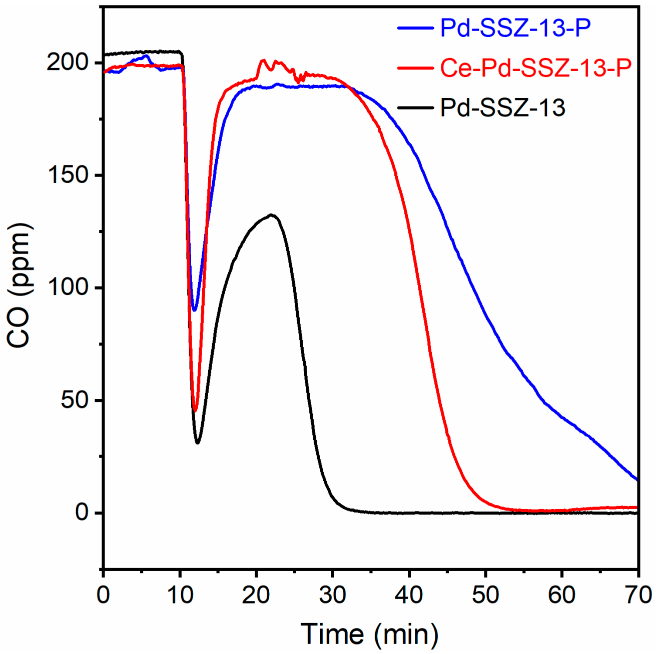

Figure S1. Concentration profiles of CO during PNA tests over Pd-SSZ-13 catalysts (gas feed: 200 ppm of NO_x, 200 ppm of CO, 3 vol.% H_2O and 10 vol.% O_2 in N_2).

Note: Prior seminal investigations have unambiguously demonstrated that hydrated ion-exchanged Pd^{2+} cationic species serve as the catalytically active moieties for low-temperature CO oxidation[1], and consequently, the intrinsic CO oxidation performance of Pd-SSZ-13 catalysts under real-world operational conditions is intrinsically dictated by the speciation and population of Pd cationic species. As elucidated in Figure S1, exposure to P species induces a marked depletion of Pd cationic species, whereas Ce modification effectively retards this detrimental deactivation pathway.

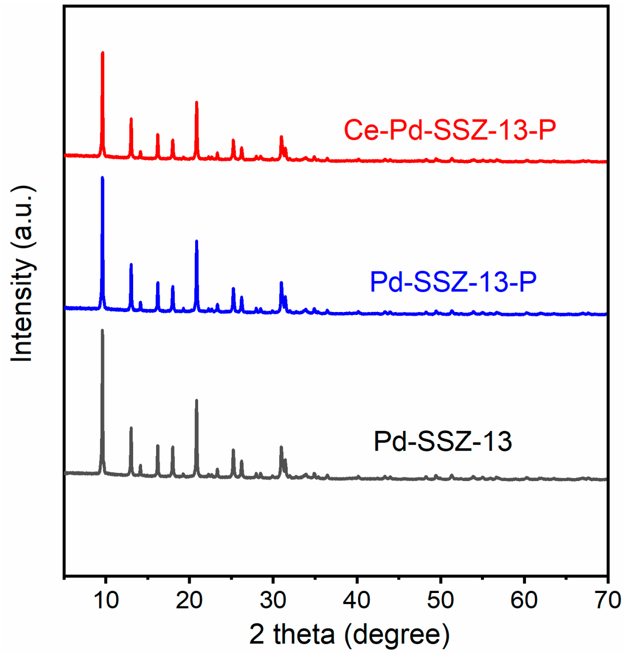

Figure S2. XRD patterns of P-poisoned Ce-Pd-SSZ-13, and Pd-SSZ-13 catalysts before and after P poisoning.

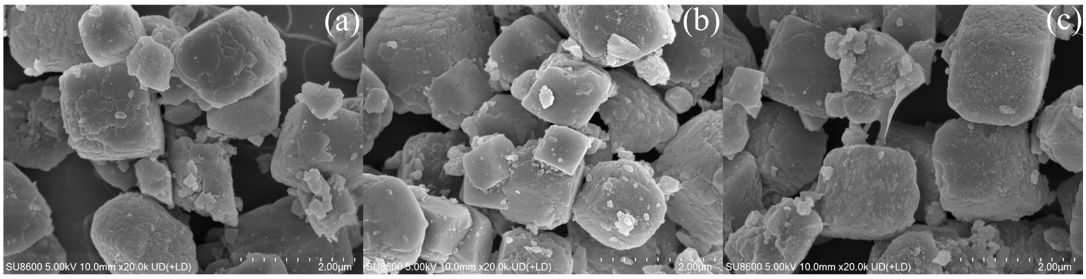

Figure S3. Representative FE-SEM images of Pd-SSZ-13 (a), Pd-SSZ-13-P (b), and Ce-Pd-SSZ-13-P (c).

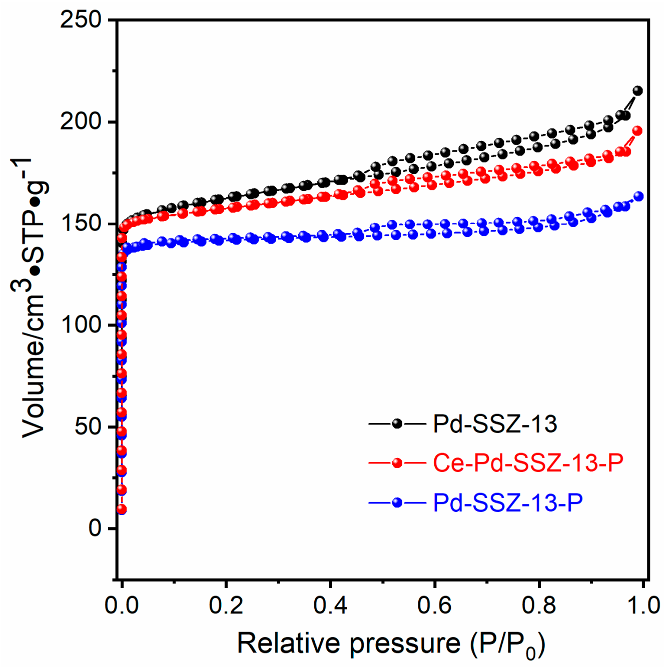

Figure S4. N_2 adsorption-desorption isotherms of Pd-SSZ-13, Pd-SSZ-13-P and Pd-SSZ-13-P catalysts.

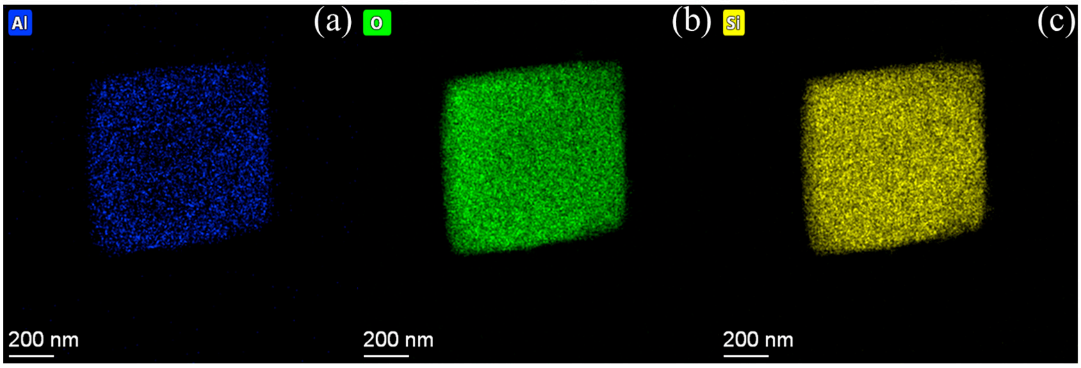

Figure S5. EDX mappings (Al, O, Si) of Pd-SSZ-13 sample.

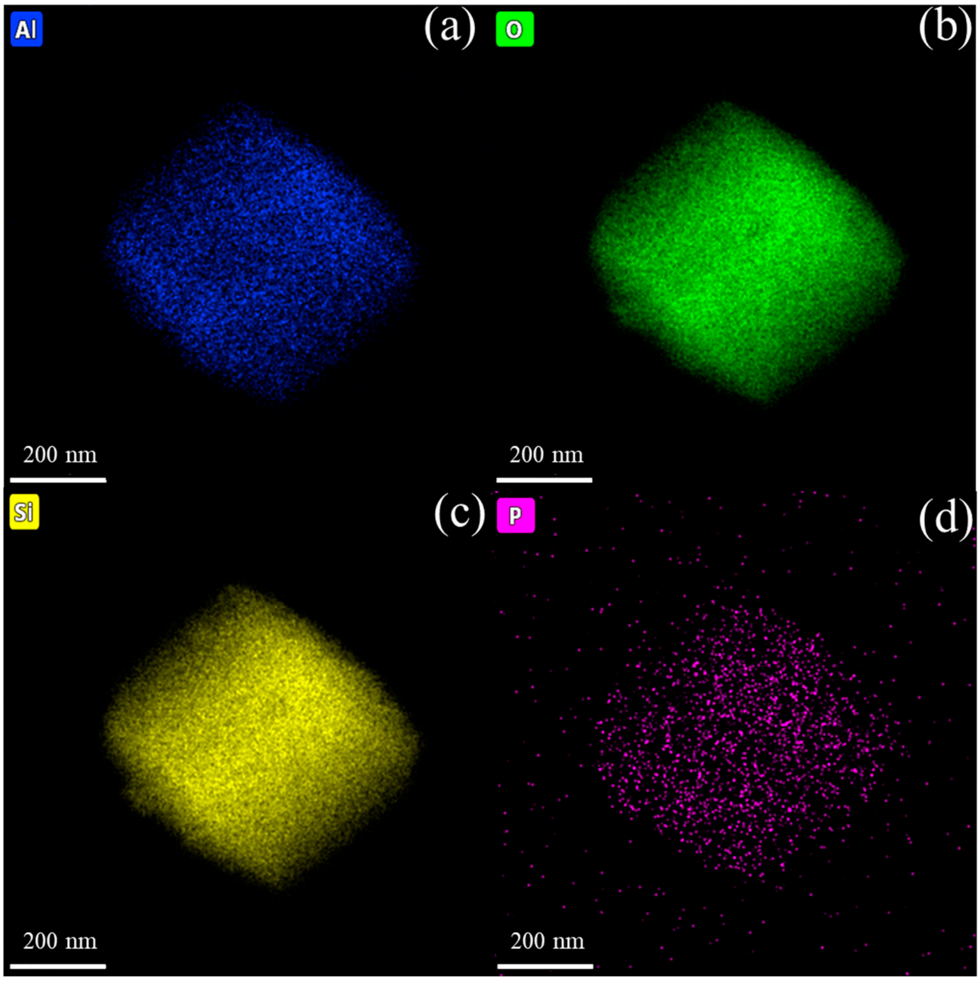

Figure S6. EDX mappings (Al, O, Si, P) of Pd-SSZ-13-P sample.

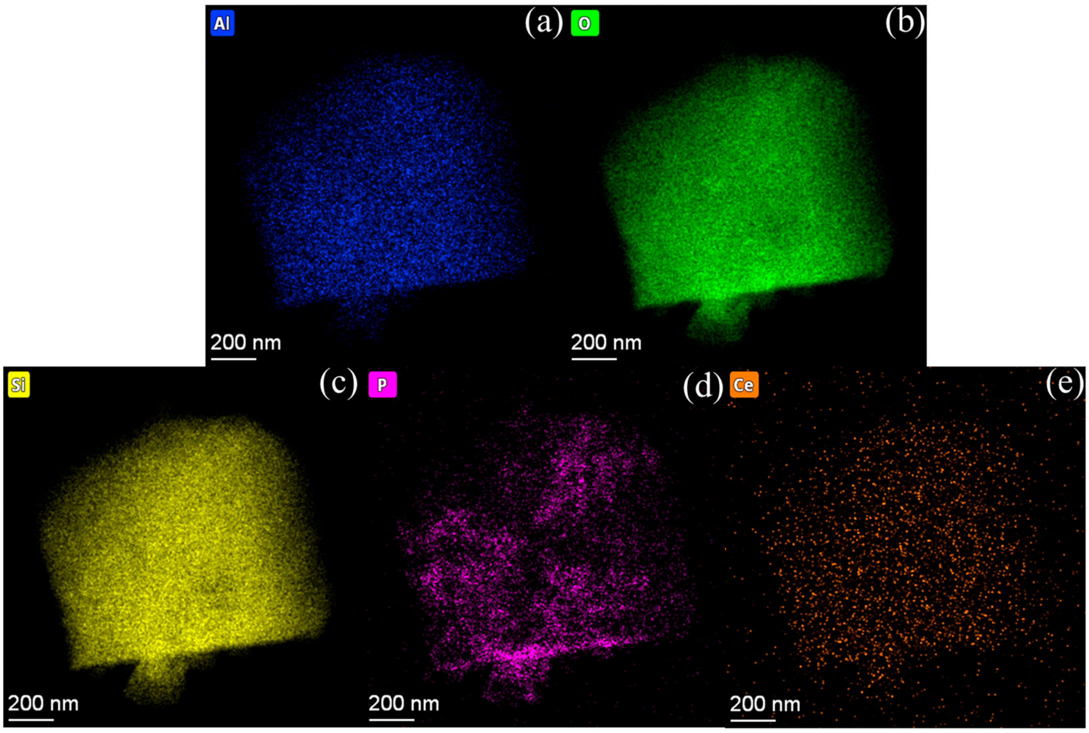

Figure S7. EDX mappings (Al, O, Si, P, Ce) of Ce-Pd-SSZ-13-P sample.

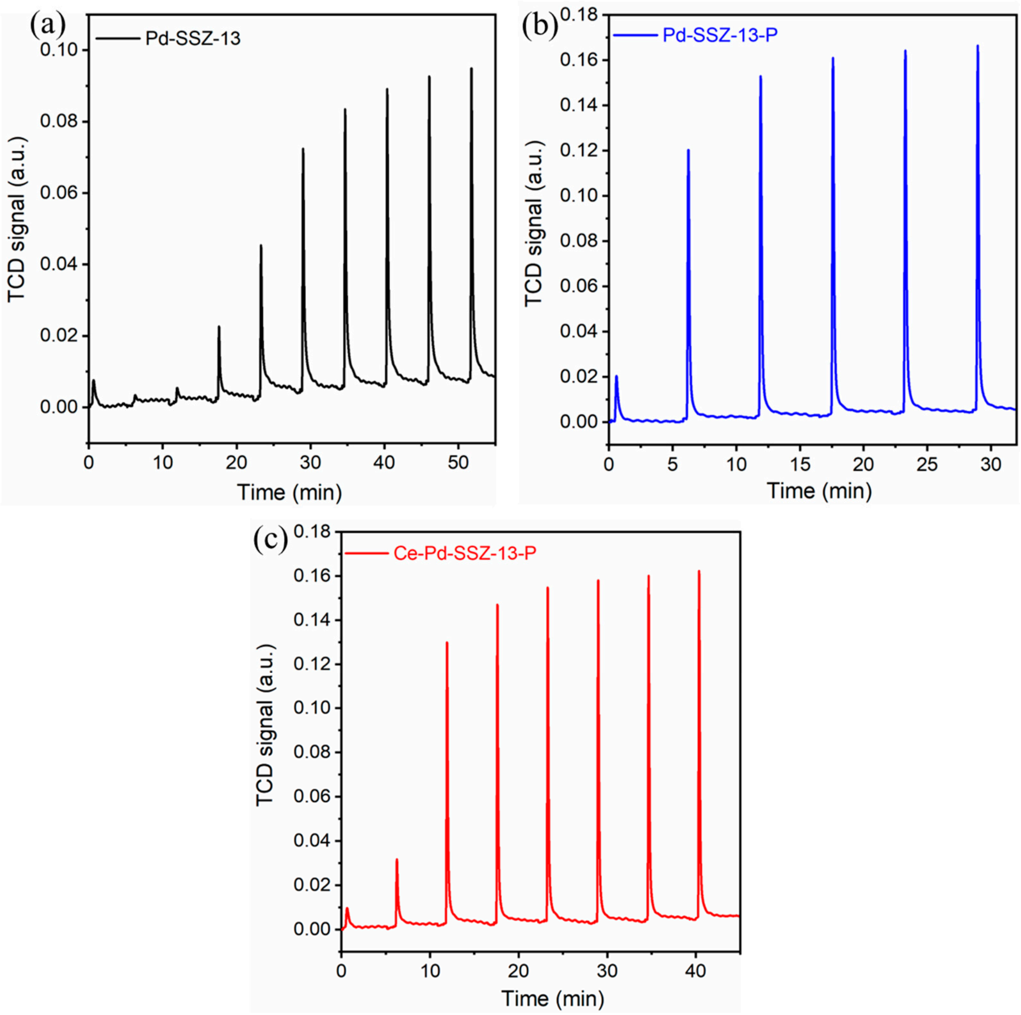

Figure S8. The profiles of CO pulse adsorption for the Pd-SSZ-13(a), P-poisoned Pd-SSZ-13-P (b) and Ce-Pd-SSZ-13-P (c) catalysts. Adsorbed CO amount and Pd dispersion for Pd-SSZ-13 samples as determined by pulse CO adsorption experiments. A CO/Pd stoichiometry of 1 was adopted to calculate the Pd dispersion.

References

[1] K. Mandal, Y. Gu, K.S. Westendorff, S. Li, J.A. Pihl, L.C. Grabow, W.S. Epling, C. Paolucci, Condition-dependent Pd speciation and NO adsorption in Pd/zeolites, ACS Catal., 10 (2020) 12801-12818.
